# On Application of the Empirical Bayes Shrinkage in Epidemiological Settings

**DOI:** 10.3390/ijerph7020380

**Published:** 2010-01-28

**Authors:** Yuejen Zhao, Andy H. Lee, Tony Barnes

**Affiliations:** 1 Institute of Advanced Studies, Charles Darwin University, Darwin NT 0909, Australia; 2 Health Gains Planning Branch, Department of Health and Families, NT 0801, Australia; E-Mail: tony.barnes@nt.gov.au; 3 School of Public Health, Curtin Health Innovation Research Institute, Curtin University of Technology, WA 6845, Australia; E-Mail: andy.lee@curtin.edu.au

**Keywords:** analysis of variance, computer simulation, reliability and validity, statistical bias, statistical data analysis

## Abstract

This paper aims to provide direct and indirect evidence on setting up rules for applications of the empirical Bayes shrinkage (EBS), and offers cautionary remarks concerning its applicability. In epidemiology, there is still a lack of relevant criteria in the application of EBS. The bias of the shrinkage estimator is investigated in terms of the sums of errors, squared errors and absolute errors, for both total and individual groups. The study reveals that assessing the underlying exchangeability assumption is important for appropriate use of EBS. The performance of EBS is indicated by a ratio statistic *f* of the between-group and within-group mean variances. If there are significant differences between the sample means, EBS is likely to produce erratic and even misleading information.

## 1. Introduction

There have been widespread interest in and applications of “shrinkage” estimators in epidemiology and demographic analysis for the purposes of smoothing spatial fluctuations, stabilizing estimates, and reducing sampling and non-sampling errors [1–4]. Prior researches have also demonstrated that the coefficient shrinkage is potentially useful for selection of epidemiological models and control of multiple confounders using modern hierarchical modeling techniques [5,6]. The term shrinkage refers to a statistical phenomenon that the posterior estimate of the prior mean is shifted from the sample mean towards the prior mean [7]. The Bayesian approach to the shrinkage estimation is to use the prior distribution and the likelihood (based on the data) to determine the posterior distribution. It has been regarded as empirical Bayes shrinkage (EBS), when there is no information for the prior, and the observed data are employed to postulate the prior distribution, assuming the sample means were drawn from the same population [8].

The shrinkage estimator was first proposed by Stein [9] in the 1950s as an alternative to the ordinary least squares (OLS) estimator *i.e.* the sample mean to produce smaller mean squared errors. In epidemiology, the EBS has been increasingly used for stabilizing disease incidence, prevalence and mortality estimates, as well as improving reliability of the estimates [10–14]. Although the underlying principles of the EBS estimator are still controversial [15–17], it is generally believed to provide an improvement over the OLS for reducing error risk in decision making [18]. Nevertheless, the EBS is subject to bias, error and arbitrary judgment [6]. Evidence also exists that this dedicated statistical technique has been misused without due considerations [15,19,20]. Recently, the Australian Bureau of Statistics applied the EBS estimator to adjust the Indigenous population estimates for Australian states and territories in an attempt to reduce standard errors, resulting in 9% and 4% reductions in the magnitude of population estimates for the states of Western Australia and Northern Territory respectively and increase of 9% for Victoria and Tasmania [21]. This methodology has substantial repercussions for Indigenous services funding allocation, and needs to be justified.

Dating back to Efron and Morris in the early 1970s, the high risk of EBS estimation has been recognized for individual parameters far from the mean of the prior distribution [22,23]. Since then, a series of improved Stein estimators have been developed to overcome the deficiency, including limited translation, positive-part and generalized Bayes estimators [e.g., 24–26], see [27] for a review of historical details. Another strategy to reduce the risk is estimation preceded by testing, known as preliminary-test estimator, to determine whether it is efficacious to shrink or not [28–32]. In epidemiological and demographic practice, these caveats appear to be largely overlooked.

In light of ongoing debate among mathematicians and statisticians on how to improve EBS and its applications, there is a lack of relevant criteria for assisting decision-making in the possible application of EBS in epidemiological settings. This paper provides empirical evidence on setting up rules for the EBS, and offers cautionary remarks concerning its applications. In the next section, the EBS is briefly reviewed and the problems concerning the EBS are specified. A statistic is proposed to determine its applicability and simulation studies are conducted to investigate and illustrate its properties. In particular, the nature of bias in the estimator is explored. Two illustrative examples are then presented, followed by discussions.

## 2. Methods

### 2.1. Empirical Bayes Estimator

Consider an ensemble of *k* group parameters *θ*_1_, *θ*_2_,...,*θ_j_*,...,*θ_k_* to be estimated with *n* independent observations *Y_j_* = (*y*_1*j*_, *y*_2*j*_,..., *y_ij_*,..., *y*_*nj*_), *j* = 1, ..., *k*, *i* = 1, ..., *n*, where *y_ij_* is normally distributed with *E*(*Y_j_*) = *θ_j_* and *Var*(*Y_j_*) = *σ*^2^. In analogy with [9], the EBS for *θ_j_* is:

(1) $$x_{j}=B\bar{\bar{y}}+(1-B){\bar{y}}_{j}$$

where $\bar{\bar{y}}=\sum_{j=1}^{k}\sum_{i=1}^{n}y_{ij}/(nk)$ is the overall sample (grand) mean, ${\bar{y}}_{j}=\sum_{i=1}^{n}y_{ij}/n$ is the sample mean for group *j* and *B* is a shrinkage factor valued between 0 and 1 inclusive. Here, *B* = 0 represents that the sample means should not be ‘shrunk’ to the grand mean, whereas *B* = 1 indicates that the sample means should be fully ‘shrunk’ to, and replaced by the grand mean. Estimation of *B* is straightforward [33,34]:

(2) $$B=\frac{(k-3)\sigma_{\bar{y}}^{2}}{\sum_{j=1}^{k}{\left({\bar{y}}_{j}-\bar{\bar{y}}\right)}^{2}},$$

where $\sigma_{\bar{y}}^{2}=\sigma^{2}/n$ is estimated by:

(3) $${\hat{\sigma }}_{\bar{y}}^{2}=\frac{\sum_{j=1}^{k}\sum_{i=1}^{n}{\left(y_{ij}-{\bar{y}}_{j}\right)}^{2}}{n(nk-k)}.$$

If the within-group mean variance is small relative to the between-group mean variance, the shrinkage factor will be small, and vice versa. An iterative estimating procedure has been developed for the unequal variance situation [34]. The EBS is believed to be an optimal combination of the sample mean and the grand mean, and increases reliability of the estimates because of its smaller sum of squared errors (*SSE*):

(4) $$SSE=\sum_{j=1}^{k}{\left({\hat{\theta }}_{j}-\theta_{j}\right)}^{2}.$$

The definition of risk by the quadratic lost function provides a useful means for risk minimization in decision making [35]. In the simulation study below, the bias (or accuracy) of the estimators will be evaluated in terms of the sum of errors (*SE*), defined as $\sum_{j=1}^{k}\left({\hat{\theta }}_{j}-\theta_{j}\right)$, the precision (or reliability) will be assessed using the *SSE* and the sum of absolute errors (*SAE*), defined as $\sum_{j=1}^{k}\left|{\hat{\theta }}_{j}-\theta_{j}\right|$, analogous to the elaboration by Hastie et al [36]. Because the task is to estimate *θ_j_*, the performance of the estimator is assessed for each *θ_j_* by:

(5) $$SE_{j}=\sum_{l=1}^{Q}\left({\hat{\theta }}_{j(l)}-\theta_{j}\right),$$

(6) $$SSE_{j}=\sum_{l=1}^{Q}{\left({\hat{\theta }}_{j(l)}-\theta_{j}\right)}^{2},$$

(7) $$SAE_{j}=\sum_{l=1}^{Q}\left|{\hat{\theta }}_{j(l)}-\theta_{j}\right|,$$

where *l* = 1,..., *Q* with *Q* being the total number of simulations. If the *SE_j_* is close to zero, the bias is small for *θ_j_*. Unlike *SSE_j_* and *SAE_j_*, the *SE_j_* can be either positive or negative.

### 2.2. Problem with the EBS Estimator

Two examples from the literature [33,34] suggested that the EBS method can produce smaller *SSE* than the sample mean, i.e., *SSE*|_*θ̂*_*j*=*x*_*j*___ < *SSE*|_*θ̂*_*j*=*ȳ*_*j*___, when the expected value of parameter *θ_j_* is assumed to be the remainder average, *y͂_j_*, where:

(8) $${\tilde{y}}_{j}=\sum_{i=n+1}^{N}y_{ij}/(N-n),$$

with the total number of observations *N>n* being finite. Referring to the basketball example [34], *N* is the total number of 82 games, *n* = 10 and *y͂_j_* is the average score for the remainder 72 games.

This opens up two questions. Firstly, what happens to the *SSE* if, instead of the remainder average, the total average *Ȳ_j_* (the final score in the examples) is used, which is really the matter of concern. The use of *y͂_j_* for the assessment standard *θ_j_* in the *SSE* equation (4) is problematic, especially when *N* is not excessively large, because when *n* → *N*, *ȳ_j_* → *Ȳ_j_* and *SSE*|_*θ̂*_*j*=*ȳ*_*j*___ → 0. Unless the assessment standard *Ȳ*_1_ = *Ȳ*_2_ = ... = *Ȳ_k_* or *B* = *0*, the EBS estimate *x_j_* will not approach *Ȳ_j_* when *n* → *N*.

Secondly, a small *SSE* does not necessarily reflect either good accuracy or high precision for all groups. This begs more questions: how are the errors distributed across groups and how will the EBS behave if *SE* and *SAE* criteria are adopted rather than *SSE*?

### 2.3. Simulation Study and Analysis of Variance

Simulation study uses computer intensive procedures to provide insights about the appropriateness and accuracy of a statistical method under particular assumptions [37]. The objectives of the simulations are (i) to see if the EBS generally outperforms the OLS; (ii) to investigate under what condition the EBS will perform better; and (iii) to explicitly demonstrate the discriminative feature of the EBS estimator in terms of bias for individual groups. A large number of simulations were undertaken with all combinations of the following parameter values being considered: *n* = 20, 40, 80; *σ*^2^ = 0.0025, 0.01, 0.04, 0.25, 1, 4, 25, 100, 400; *N* = 100; *k* = 9; *j* = 1,...,9; *θ_j_* = *j*/10, *j*, 10*j; y_ij_* ~ *Normal* (*θ_j_*, *σ*^2^). These settings are devised to cover a wide range of possible combinations of differences between within-group and between-group variances. The OLS was chosen for comparison partly because of the ease of computation and partly because the OLS corresponds to the maximum likelihood estimator under a normal distribution, which is common in epidemiological settings. In the simulations, $\sigma_{\bar{y}}^{2}$ is always estimated by ${\hat{\sigma }}_{\bar{y}}^{2}$, even though *σ*^2^ is known.

The performance of *x_j_* is then analysed using the ratio *f* of the between-group mean variance and the within-group mean variance:

(9) $$f=\frac{n\sum_{j=1}^{k}{\left({\bar{y}}_{j}-\bar{\bar{y}}\right)}^{2}/(k-1)}{\sum_{j=1}^{k}\sum_{i=1}^{n}{\left(y_{ij}-\bar{y}\right)}^{2}/(nk-k)}.$$

Suppose the posterior mean $\theta_{j}∼Normal(\vartheta ,\sigma_{\theta_{j}}^{2})$ with ϑ = *E*(*θ_j_*) and $\sigma_{\theta_{j}}^{2}=\sigma^{2}/n$. Dividing both the numerator and the denominator of *f* by *σ*^2^ > 0 yields:

(10) $$\sum_{j=1}^{k}{\left({\bar{y}}_{j}-\bar{\bar{y}}\right)}^{2}/(\sigma^{2}/n)∼\chi^{2}(k-1)$$

as given by Everson [34], and:

(11) $$\sum_{j=1}^{k}\sum_{i=1}^{n}{\left(y_{ij}-\bar{y}\right)}^{2}/\sigma^{2}∼\chi^{2}(nk-k),$$

which further leads to the ratio statistic:

(12) $$f=(k-3)/[(k-1)B]∼F_{\left(k-1,nk-k\right)}.$$

This statistic is similar in spirit to Sclove, Morris and Radhakrishnan [29]. Note that the *f* statistic is inversely proportional to *B*.

## 3. Results

### 3.1. Simulations

The number of replications *Q* is set to 1,000, which is considered sufficient (>500) for detecting a 0.02 permissible difference (one-fifth of the difference between the minimum group means), given the variance 0.25, *n* = 20, type I error 0.05 and the power 0.95 [37]. The first part of the study is to compare *SSE* and *SAE* of the EBS estimator with those of the OLS estimator. Note that *SE* is excluded because *SE|*_*θ̂*_*j*=*x*_*j*___ ≡ *SE|*_*θ̂*_*j*=*ȳ*_*j*___. The proportions of the 1,000 simulations for which *SSE* of the EBS estimator is smaller than its OLS counterpart are recorded in Table 1. The simulation results show that the EBS estimator can outperform the OLS estimator (proportion > 50%) when the parameter *θ_j_* and the remainder average *y͂_j_* are used for assessment when *σ*^2^ is large and the differences between sample means are small (*θ_j_* = *j*/10 or *θ_j_* = *j*). The EBS estimator, however, performs slightly worse than the OLS estimator when the total mean *Ȳ_j_* is used for assessment and *n* is large, and particularly when *σ*^2^ is large. The performance of *x_j_* appears to be related to both *σ*^2^ and variance between sample means $\sigma_{\theta_{j}}^{2}=Var(\theta_{j})$. It does not outperform the OLS estimator when *σ*^2^ is small relative to $\sigma_{\theta_{j}}^{2}$.

It is evident that the performance of *x_j_* closely relates to the *f* value. If the group *θ_j_* ’s were equal, *f* would be small and the between-group mean variance would be close to the within-group mean variance. The simulation results show that when *f* is small, the EBS estimator is more likely to outperform the OLS estimator. In the baseball example of Morris [33], the EBS estimator performed well, because *f* = 1.12 did not exceed the *F* distribution 5% cut-off value of 1.64. In contrast, if the group *θ_j_* ’s were not equal, the between-group mean variance would be large (relative to the within-group variance), and thus would inflate the *f* value. If *f* is large, for example, *f* > *F*_0.05 (*k*−1,*nk*−*k*)_, the EBS estimator will not outperform the OLS estimator in terms of *SSE* criteria. This implies that the underlying exchangeability assumptions of the EBS do not hold and the group means should not be shrunk. Table 1 lists the *f* values when *n* → ∞. The results confirm that when *f* < 1.94 (*F*_0.05(8,∞)_), the EBS estimator performs better than the OLS estimator, i.e., the proportion of *SSE|*_*θ̂*_*j*=*x*_*j*___ *< SSE|*_*θ̂*_*j*=*ȳ*_*j*___ is much greater than 50%. Simulation results for *SAE* are broadly consistent with *SSE* results and not presented for brevity.

**Figure 1.**
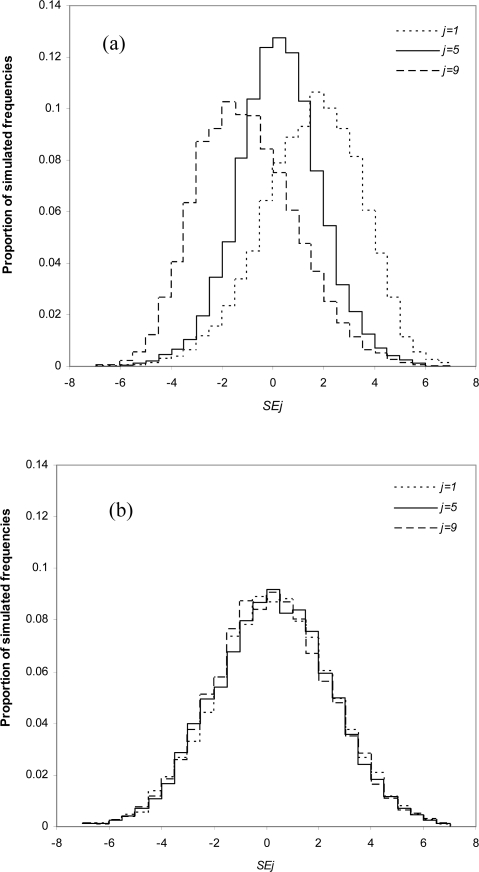
Error distribution for groups with minimum, medium and maximum values. (a) Sum of Errors (*SE_j_*) for *j* = 1, 5, 9 for Empirical Bayes Shrinkage Estimates; (b) Sum of Errors (*SE_j_*) for *j* = 1, 5, 9 for Ordinary Least Square Estimates.

The errors are next assessed for individual *θ_j_* in the second part of the simulation study. The individual *SE_j_*, *SSE_j_* and *SAE_j_* analyses unveil some undesirable features of the EBS estimator. Table 2 shows that the EBS estimator has a positive bias for groups with sample means far below the grand mean, for example, *j* = 1. Meanwhile, the EBS estimator tends to have a negative bias for groups with sample means far above the grand mean, for example, *j* = 9. The EBS estimator introduces a statistical bias towards the grand mean, which is skewed against marginal values. This is clearly illustrated in the results of the simulations shown in Figure 1. Panel (a) of Figure 1 shows that the *SE*_1_ for EBS estimate *x*_1_ is skewed positively, the *SE*_5_ for *x*_5_ has a symmetric distribution, whereas *x*_9_ is skewed negatively. By comparison, panel (b) clearly indicates that regardless of the magnitude of the means, the distributions of *SE_j_* for all three OLS estimators *ȳ*_1_, *ȳ*_5_ and *ȳ*_9_ are overlapping and symmetrical. These plots confirm the presence of bias in the EBS estimator and the lack of bias in the OLS estimator. Furthermore, this bias from EBS is negatively correlated with the marginal position of the parameter in relation to other parameters.

Table 3 presents the *SSE_j_* by groups. It is evident that the EBS estimator performs well under certain conditions corresponding to the top-right corner of Table 3 (*σ*^2^ = 100; *θ_j_* = 0.1, 0.5, 0.9; *f* = 0.015), where the EBS *SSEj* is smaller than the OLS *SSEj*. As is shown in most other cases of Table 3, for groups with value far away from the grand mean (e.g., *j* = 1, 9), the EBS *SSE_j_* is larger than the OLS *SSE_j_*. For groups with value close to the grand mean (e.g., *j* = 5), the EBS *SSE_j_* is smaller than or equal to the OLS *SSE_j_*. The results indicates that the EBS estimator reallocates sum of squared errors unevenly across the groups, less for the central values and more for the minimum and maximum values. Again, simulation results for individual *SAE_j_* are generally in agreement with those for *SSE_j_* and thus are omitted for brevity.

In view of the above results, the EBS estimator may not increase the reliability of the estimates. When *f* is small, the EBS estimator can increase the reliability more for those means close to the grand mean, but less for those means far away from the grand mean. When *f* is large, the EBS estimator actually decreases the reliability especially for the means very different from the grand mean. The overall smaller *SSE* for which the EBS is designed does not necessarily lead to an increase in precision for all groups. It is very likely for the marginal groups that the EBS will produce both greater bias and less precision if the *f* value is large. When *f* exceeds *F*_0.05(*k*–1,*nk*–*k*)_, the EBS estimator ceases to be preferable to the OLS estimator given the statistical bias introduced. In this case, potential confounder(s) need to be identified, and further divisions of ensembles or stratifications are necessary to ensure the *f* value is not exceedingly large when EBS is applicable.

### 3.2. Examples

Two examples using real data are provided below to demonstrate instances where the OLS estimators generate a lower *SSE* than the EBS estimators. In both these examples the inadvisability of using the EBS estimator is suggested by the *f* statistic criterion.

#### Example 1: Mumps

The first application concerns mumps notifications per 100,000 by State/Territory from the Australian National Notifiable Diseases Surveillance System [38]. The data from 2001 to 2007 are taken to predict the 2008 notification rate, and the year-to-date 2008 notification rate is used to evaluate the EBS estimate; see Table 4. Suppose the notification rates follow a normal distribution and the EBS is applicable. Because of the difference in population size between State/Territories, unequal variances are considered appropriate and the shrinkage factors are estimated iteratively [34]. The estimated shrinkage factors and corresponding EBS estimates for the 2008 notification rates by State/Territory are listed at the bottom rows of Table 4. The *SSE* for the EBS estimator is 267.6, much greater than the *SSE* of 202.5 for the OLS estimator. The EBS estimators do not provide better estimates than the OLS estimators (in terms of *SSE*) in this situation. Here *f* = 13.09 is much greater than *F*_0.05(7,56)_ = 2.18 and therefore the EBS estimator is not recommended.

#### Example 2: Birth Weights

The birth weight data are taken from the perinatal data collections from 2003 to 2007 in the Northern Territory, Australia. There are seven districts in the Northern Territory, namely Alice Springs Rural, Alice Springs Urban, Barkly, Darwin Rural, Darwin Urban, East Arnhem and Katherine. The annual average birth weights from 2003 to 2006 are used to estimate the true average birth weight for each region over the period 2003–2007, as shown in Table 5. The *f* value of 34.30 is much greater than *F*_0.05(6,21)_ = 2.57, and the EBS performed badly with *SSE* = 648, much greater than the *SSE* = 601 of the OLS estimator. Then we stratify the birth weights by identifying and separating out non-Aboriginal infants. The *f* value decreases to 3.71, indicating the performance of the EBS estimator has improved substantially. In accordance with the *f* statistic criterion, the EBS is still not applicable after stratification, indicating further potential confounders (such as rurality) may operate. Due to the small number of districts, further division of the ensemble based on rurality is not performed.

In the above two examples, the relative merits of the EBS and OLS estimators are reversed compared with the sport examples advocating the EBS estimator [33,34].

## 4. Discussion

The EBS estimator is sometimes considered as a possible solution to the problem of unstable estimates and a way to reduce standard errors. This study demonstrates that when the variance ratio statistic *f* is large, the EBS estimator offers little reduction in standard errors for all groups, but instead it can potentially increase standard errors and bias for marginalized groups.

The EBS rests on some important implicit assumptions such as unimodal probability distribution and exchangeability [17]. To make the assumptions explicit, for the EBS to be valid, the groups within each ensemble have to be “similar”, exchangeable random quantities from the same prior bell-shaped distribution. If the *f* value indicates that they are unlikely similar groups from the same distribution, then the underlying assumptions are violated. A remedy to this problem is to stratify or partition the data into credible ensembles according to confounders in order to satisfy these assumptions. In doing so, each ensemble will have its own model prior distribution with little between-group heterogeneity relative to within-group sampling error. Alternatively, if additional covariate or potential confounder information is available, hierarchical regression, multilevel model or mixed model appear more appropriate to allow the prior parameters to vary at more than one level and enable structural prior information to be incorporated into parameter estimates [39–41]. The multivariate coefficient shrinkage, rather than EBS, seems to be the answer to address the confounding and collinearity issues [5]. Forcing EBS without consideration of exchangeability may lead to loss of most of the statistical gains [42].

The rationale behind shrinkage was to minimize the risk by considering a prescribed loss function, rather than unbiased estimation for the parameter. The improvement in the risk is significant if the individual components are close to the point towards which these estimators shrink and the ensemble point estimator is of primary interest [23]. Many authors have contributed to improving both ensemble and individual properties for the shrinkage estimators, including the preliminary test approach [29–31,43]. The main advantage of the EBS estimator is a sacrifice of unbiasedness for improved precision. The *f* value plays a role in suggesting those situations under which this trade-off is beneficial and those under which it is not. When *f* becomes large, the benefits of improved precision appear to be diminishing and offset by unacceptably large bias and a greater degree of volatility for marginal groups. This process can be interpreted as a preliminary test for exchangeability. At first, the null hypothesis *θ*_1_ = *θ*_2_ = ... = *θ_k_* is tested with the *f* statistic. If *f* > *F*_*α*(*k*–1,*nk*–*k*)_, the hypothesis is rejected at the significance level α, *θ_j_’s* are not really exchangeable and EBS is not indicated to be suitable.

Epidemiologists and practitioners may not be fully aware of the possible problematic and differential nature of both bias and volatility resulting from EBS estimation; with benefits being directed towards the ones having a large population while disadvantaging those having a small population and sample size. Such differential shrinkage is often counter-intuitive. The arbitrary and unjustified shrinkage may be regarded as unfair or merely data manipulation by those being evaluated, especially when the precisions for individual group estimates are of equal interest, as distinct from the general research situation when the overall precision is of primary interest.

In summary, the purpose of the EBS estimator is to reduce “risk” in terms of *SSE*. To apply the EBS estimator appropriately, epidemiologists need to assess the underlying exchangeability assumption. If there are significant differences between the sample means, EBS is likely to produce erratic and even misleading information.

## Figures and Tables

**Table 1.** Proportions (%) of *SSE|*_*θ̂*_*j*=*x*_*j*___ < *SSE*|_*θ̂*_*j*=*ȳ*_*j*___ based on *Q* = 1000 replications.

	*n*	**Assessing standard**	*σ*^2^						
			0.0025	0.01	0.04	1	25	100	400
*θ* =*j*/10									

*f*			600.0	150.0	37.50	1.500	0.060	0.015	0.004

	20	*θ_j_*	50.4	54.4	61.7	82.5	93.9	93.0	94.2
		*Ȳ_j_*	49.3	52.5	59.8	74.4	87.5	84.6	85.2
		*v͂*	49.6	53.4	61.5	79.7	92.5	90.3	91.0
	40	*θ_j_*	51.1	52.9	56.1	79.7	93.7	93.2	93.7
		*Ȳ_j_*	51.7	50.7	54.3	61.5	74.1	71.9	68.9
		*v͂*	52.1	52.0	56.1	73.1	89.6	88.3	87.8
	80	*θ_j_*	52.2	50.9	54.0	74.1	92.6	92.0	93.9
		*Ȳ_j_*	52.4	48.2	50.0	50.3	49.6	47.0	48.7
		*v͂*	53.5	49.4	53.6	66.6	83.9	83.3	82.7

*θ* =*j*									

*f*			60 000	15 000	3750	150.0	6.000	1.500	0.375

	20	*θj*	50.4	54.4	61.7	49.7	69.7	83.9	90.7
		*Ȳ_j_*	49.3	52.5	59.8	49.8	63.5	75.3	82.3
		*v͂*	49.6	53.4	61.5	49.9	68.1	80.8	88.7
	40	*θ_j_*	51.1	52.9	56.1	50.1	66.5	77.6	86.2
		*Ȳ_j_*	51.7	50.7	54.3	50.8	56.8	60.8	65.2
		*v͂*	52.1	52.0	56.1	50.9	62.8	73.0	80.2
	80	*θ_j_j*	52.2	50.9	54.0	48.5	62.9	71.2	85.5
		*Ȳ_j_*	52.4	48.2	50.0	51.0	46.7	48.8	49.1
		*v͂*	53.5	49.4	53.6	51.0	54.5	65.4	74.2

*θ* = 10*j*									

*f*			6 000 000	1 500 000	375 000	15 000	600.0	150.0	37.50

	20	*θ_j_*	49.9	49.0	49.3	49.7	52.9	54.5	60.2
		*Ȳ_j_*	49.3	50.3	49.8	49.8	50.9	51.9	56.5
		*v͂*	49.3	50.3	49.8	49.9	51.3	52.6	57.8
	40	*θ_j_*	48.4	49.0	49.5	50.1	52.1	53.0	54.5
		*Ȳ_j_*	50.7	48.9	49.1	50.8	51.0	51.8	51.2
		*v͂*	50.7	48.9	49.2	50.9	51.8	53.2	53.6
	80	*θ_j_*	50.1	49.3	49.3	48.5	52.7	52.2	54.6
		*Ȳ*	50.2	50.0	50.3	51.0	49.7	50.3	47.3
		*v͂*	50.2	50.0	50.3	51.0	50.8	52.2	51.1

**Table 2.** The sum of errors (100*SE_j_*) by groups with *j* = 1, 5, 9

*n*		*σ*^2^	0.01			1			100		
			
		*j*	1	5	9	1	5	9	1	5	9
		*θ_j_*	0.1	0.5	0.9	0.1	0.5	0.9	0.1	0.5	0.9
20	*ȳ_j_*		−0.008	0.037	−0.007	0.786	−0.200	−0.227	8.479	−6.425	1.004
	*x_j_*		0.191	0.037	−0.205	13.475	−0.219	−12.996	29.164	3.062	−24.211
40	*ȳ_j_*		0.019	0.016	−0.019	0.510	0.578	0.321	3.751	−7.531	0.067
	*x_j_*		0.119	0.016	−0.119	8.204	0.483	−7.498	28.719	−1.757	−28.441
80	*ȳ_j_*		−0.012	0.017	−0.011	0.271	0.161	−0.017	−1.264	−5.765	−0.045
	*x_j_*		0.037	0.017	−0.061	4.683	0.170	−4.397	29.172	−1.436	−30.025

		*θ_j_*	1	5	9	1	5	9	1	5	9
20	*ȳ_j_*		0.023	0.039	−0.080	0.871	−0.426	−0.976	1.489	4.195	0.162
	*x_j_*		0.043	0.039	−0.100	2.856	−0.425	−2.961	129.271	2.284	−126.039
40	*ȳ_j_*		−0.013	0.046	−0.041	0.515	−0.934	−0.286	3.159	3.994	−3.299
	*x_j_*		−0.003	0.046	−0.051	1.513	−0.932	−1.283	82.075	2.813	−82.005
80	*ȳ_j_*		−0.040	−0.002	−0.036	0.142	−0.924	−0.200	2.332	−0.916	−1.153
	*x_j_*		−0.035	−0.002	−0.041	0.641	−0.923	−0.699	46.580	−0.904	−45.703

		*θ_j_*	10	50	90	10	50	90	10	50	90
20	*ȳ_j_*		−0.089	−0.008	−0.025	−0.956	0.198	−0.331	2.691	8.139	−3.101
	*x_j_*		−0.087	−0.008	−0.027	−0.756	0.198	−0.531	22.794	8.100	−23.191
40	*ȳ_j_*		−0.092	−0.083	−0.083	−0.772	−0.084	−0.298	−1.020	7.342	−3.903
	*x_j_*		−0.091	−0.083	−0.084	−0.673	−0.084	−0.398	8.977	7.322	−13.890
80	*ȳ_j_*		−0.066	−0.020	−0.030	−0.496	−0.012	−0.293	−1.948	4.893	−6.302
	*x_j_*		−0.065	−0.020	−0.030	−0.447	−0.012	−0.343	3.043	4.886	−11.284

**Table 3.** The sum of squared errors (100*SSE_j_*) by groups with *j* = 1, 5, 9.

*n*		*σ*^2^	0.01			1			100		
			
		*j*	1	5	9	1	5	9	1	5	9
		*θ_j_*	0.1	0.5	0.9	0.1	0.5	0.9	0.1	0.5	0.9
20	*ȳ_j_*		0.0479	0.0497	0.0477	5.0400	5.1221	4.8391	509.64	502.83	462.32
	*x_j_*		0.0482	0.0493	0.0479	5.5647	2.8272	5.2105	181.62	178.89	186.64
40	*ȳ_j_*		0.0248	0.0244	0.0245	2.6060	2.4557	2.5326	248.01	256.36	247.09
	*x_j_*		0.0249	0.0242	0.0246	2.8567	1.7153	2.6752	95.284	86.476	94.234
80	*ȳ_j_*		0.0128	0.0119	0.0128	1.2761	1.1932	1.2172	119.23	123.92	111.17
	*x_j_*		0.0128	0.0119	0.0128	1.3926	0.9717	1.3115	49.919	40.804	52.251

		*θ_j_*	1	5	9	1	5	9	1	5	9
20	*ȳ_j_*		0.0517	0.0491	0.0510	5.0175	5.2305	5.4695	485.60	495.86	530.51
	*x_j_*		0.0517	0.0491	0.0511	5.0747	5.1849	5.5320	537.33	267.53	548.56
40	*ȳ_j_*		0.0244	0.0249	0.0271	2.5130	2.6806	2.8059	233.65	241.30	239.03
	*x_j_*		0.0244	0.0249	0.0271	2.5281	2.6687	2.8173	265.57	162.31	269.45
80	*ȳ_j_*		0.0128	0.0125	0.0137	1.2054	1.3085	1.3438	118.80	129.07	119.67
	*x_j_*		0.0128	0.0125	0.0138	1.2080	1.3055	1.3471	130.91	104.15	132.15

		*θ_j_*	10	50	90	10	50	90	10	50	90
20	*ȳ_j_*		0.0479	0.0486	0.0469	4.7860	4.9156	5.0841	501.95	497.18	503.44
	*x_j_*		0.0479	0.0486	0.0469	4.7825	4.9152	5.0854	504.31	492.75	507.06
40	*ȳ_j_*		0.0255	0.0261	0.0229	2.5097	2.5017	2.6234	253.43	243.67	251.46
	*x_j_*		0.0255	0.0261	0.0229	2.5082	2.5016	2.6240	253.71	242.56	252.78
80	*ȳ_j_*		0.0120	0.0132	0.0120	1.2461	1.2779	1.2834	126.39	116.47	118.15
	*x_j_*		0.0120	0.0132	0.0120	1.2456	1.2779	1.2837	126.36	116.21	118.92

**Table 4.** Australian mumps notification rate (per 100,000 population).

	State/Territory*							
	**ACT**	**NSW**	**NT**	**Qld**	**SA**	**Tas**	**Vic**	**WA**
**2001**	0.3	0.4	0.5	0.1	0.8	0.4	0.8	1.5
**2002**	0	0.4	0.5	0.2	0.7	0	0.2	0.7
**2003**	0.6	0.5	0	0.3	0.8	0	0.1	0.7
**2004**	0.9	1	0	0.4	0.3	0	0.1	0.5
**2005**	0.3	1.6	3.4	1.7	0.5	0	0.4	1.1
**2006**	0.3	2.3	3.3	1.4	1.3	0	0.3	0.8
**2007**	1.2	4.7	28.8	1.1	1.4	0.4	0.3	5.2
**2008 (year-to-date)**	0	1	19.1	0.6	0.9	0.4	0.3	4.5

*ȳ_j_*	0.5	1.6	5.2	0.7	0.8	0.1	0.3	1.5
*B_j_*	0.11	0.01	0.17	0.01	0.03	0.08	0.01	0.02
*x_j_*	0.6	1.6	4.6	0.7	0.8	0.2	0.3	1.5

* ACT: Australian Capital Territory; NSW: New South Wales; NT: Northern Territory; Qld: Queensland; SA: South Australia; Tas: Tasmania; Vic: Victoria; WA: Western Australia.

**Table 5.** Birth weights (grams) by regions, 2003–2007, Northern Territory, Australia.

		District*							**Total**
		**ASR**	**ASU**	**BD**	**DR**	**DU**	**EA**	**KD**	
**NT**^†^									
**2003–2006**	*ȳ*_*j*_	3,182	3,381	3,137	3,058	3,326	3,121	3,186	3,198
	SD^‡^	33.3	27.1	72.5	26.3	8.0	27.4	46.8	113.8
**2003–2007**	*θ_j_*	3,187	3,386	3,145	3,051	3,331	3,141	3,189	
	*x_j_*	3,182	3,377	3,139	3,060	3,323	3,122	3,186	

**NT non-Aboriginal**									
**2003–2006**	*ȳ_j_*	3,494	3,421	3,322	3,324	3,347	3,504	3,320	3,390
	SD^‡^	119.8	25.9	162.7	33.6	8.7	73.8	53.1	108.0
**2003–2007**	*θ_j_*	3,494	3,433	3,371	3,324	3,351	3,494	3,324	
	x*_j_*	3,476	3,415	3,335	3,336	3,355	3,484	3,333	

* ASR: Alice Springs Rural; ASU: Alice Springs Urban; BD: Barkly District; DR: Darwin Rural; DU: Darwin Urban; EA: East Arnhem; KD: Katherine District;

† NT: Northern Territory;

‡ SD: standard deviation.

## References

[1] Efron B Morris C Data analysis using Stein’s estimator and its generalisation J. Am. Stat. Assoc 1975 70 311 319

[2] Steinberg J Synthetic Estimates for Small Areas: Statistical Workshop Papers and Discussion Department of Health, Education and Welfare Rockville, MD, USA 1979

[3] Clayton D Kaldor J Empirical Bayes estimates of age-standardized relative risks for use in disease mapping Biometrics 1987 43 671 681 3663823

[4] Castner LA Schirm AL Empirical Bayes Shrinkage Estimates of State Food Stamp Participation Rates for 1998–2000 Mathematica Policy Research Princeton, NJ, USA 2003

[5] Greenland S Invited commentary: variable selection versus shrinkage in the control of multiple confounders Am. J. Epidemiol 2008 167 523 529 18227100 10.1093/aje/kwm355

[6] Rothman KJ Greenland S Lash TL Modern Epidemiology3rd ed Lippincott Williams & Wilkins Philadelphia, PA, USA 2008

[7] Armitage P Berry G Matthews JNS Statistical Methods in Medical Research4th ed Blackwell Publishing London, UK 2002

[8] Efron B Morris C Stein’s estimation rule and its competitors-an empirical Bayes approach J. Am. Stat. Assoc 1973 68 117 130

[9] Stein C Inadmissibility of the usual estimator for the mean of a multivariate normal distribution Proceedings of the Third Berkeley Symposium on Mathematical Statistics and Probability University of California Press Berkeley, CA, USA 1956 1 197 208

[10] Casper M Wing S Strogatz D Davis CE Tyroler HA Antihypertensive treatment and US trends in stroke mortality, 1962 to 1980 Am. J. Public Health 1992 82 1600 1606 1456333 10.2105/ajph.82.12.1600 PMC1694550

[11] Cislaghi C Biggeri A Braga M Lagazio C Marchi M Exploratory tools for disease mapping in geographical epidemiology Stat. Med 1995 14 2363 2381 8711275 10.1002/sim.4780142108

[12] Chambless LE Folsom AR Clegg LX Sharrett AR Nieto FJ Shahar E Rosamond W Evans G Carotid wall thickness is predictive of incident clinical stroke Am. J. Epidemiol 2000 151 478 487 10707916 10.1093/oxfordjournals.aje.a010233

[13] Beckett LA Tancredi DJ Parametric empirical Bayes estimates of disease prevalence using stratified samples from community populations Stat. Med 2000 19 681 695 10700739 10.1002/(sici)1097-0258(20000315)19:5<681::aid-sim343>3.0.co;2-y

[14] Graham P Intelligent smoothing using hierarchical Bayesian models Epidemiology 2008 19 493 495 18414089 10.1097/EDE.0b013e31816b7859

[15] Carlin JB Louis TA Bayes and Empirical Bayes Methods for Data Analysis2nd ed Chapman & Hall New York, NY, USA 2000

[16] Gutmann S Stein’s Paradox is impossible in problems with finite sample space Ann. Stat 1982 10 1017 1020

[17] Greenland S Poole C Empirical-Bayes and semi-Bayes approaches to occupational and environmental hazard surveillance Arch. Environ. Health 1994 49 9 16 8117153 10.1080/00039896.1994.9934409

[18] Fabozzi FJ Kolm PN Pachamanova D Focardi SM Robust Portfolio Optimization and Management John Wiley & Sons Hoboken, NJ, USA 2007

[19] Perlman MD Chaudhuri S Reversing the Stein Effect University of Washington Seattle, WA, USA 2005 Available online: http://www.stat.washington.edu/research/reports/2005/(accessed on December 8 2008).

[20] Tate RL A cautionary note on shrinkage estimates of school and teacher effects Florida J. Educ. Res 2004 42 1 21

[21] Experimental Estimates of Aboriginal and Torres Strait Islander Australians, 2006 Australian Bureau of Statistics Canberra, Australia 2008

[22] Efron B Morris C Limiting the risk of Bayes and empirical Bayes estimators—Part 1: The Bayes case J. Am. Stat. Assoc 1971 66 807 815

[23] Efron B Morris C Limiting the risk of Bayes and empirical Bayes estimators—Part 2: The empirical Bayes case J. Am. Stat. Assoc 1972 67 130 139

[24] Lin P Tsai H Generalized Bayes minimax estimators of the multivariate normal mean with unknown covariance matrix Ann. Stat 1973 1 142 145

[25] Stein CM Estimation of the mean of a multivariate normal distribution Ann. Stat 1981 6 1135 1151

[26] Yi-Shi Shao P Strawderman WE Improving on the James-Stein positive-part estimator Ann. Stat 1994 22 1517 1538

[27] Hoffmann K Stein estimation—a review Stat. Pap 2000 41 127 158

[28] Sclove SL Improved estimators for coefficients in linear regression J. Am. Stat. Assoc 1968 63 596 606

[29] Sclove SL Morris C Radhakrishnan R Non-optimality of preliminary-test estimators for the mean of a multivariate normal distribution Ann. Math. Stat 1972 43 1481 1490

[30] Sen PK Saleh AKME On preliminary test and shrinkage M-estimation in linear models Ann. Stat 1987 15 1580 1592

[31] Khan S Saleh AKME On the comparison of the pre-test and shrinkage M-estimation in linear models Stat. Pap 2001 42 451 473

[32] Saleh AKME Theory of Preliminary Test and Stein-type Estimation with Applications Wiley New York, NY, USA 2006

[33] Morris C Parametric empirical Bayes inference: theory and applications J. Am. Stat. Assoc 1983 78 47 55

[34] Everson P A statistician reads the sports pages, Stein’s paradox revisited Chance 2007 20 49 56

[35] Gruber MHJ Improving Efficiency by Shrinkage: The James-Stein and Ridge Regression Estimators Marcel Dekker New York, NY, USA 1998

[36] Hastie T Tibshirani R Friedman JH The Elements of Statistical Learning: Data Mining, Inference, and Prediction Springer New York, NY, USA 2001

[37] Burton A Altman DG Royston P Holder RL The design of simulation studies in medical statistics Stat. Med 2006 25 4279 4292 16947139 10.1002/sim.2673

[38] Australian National Notifiable Diseases Surveillance System, 2001–2008 Australian Department of Health and Ageing Canberra, Australia Available online: http://www9.health.gov.au/cda/Source/Rpt_4.cfm (accessed on November 20 2008).

[39] Witte JS Greenland S Simulation study of hierarchical regression Stat. Med 1996 15 1161 1170 8804145 10.1002/(SICI)1097-0258(19960615)15:11<1161::AID-SIM221>3.0.CO;2-7

[40] Greenland S When should epidemiologic regressions use random coefficients? Biometrics 2000 56 915 921 10985237 10.1111/j.0006-341x.2000.00915.x

[41] West BT Welch KB Galecki AT Linear Mixed Models: A Practical Guide Using Statistical Software Chapman Hall/CRC Boca Raton, FL, USA 2006

[42] Berger JO Statistical Decision Theory and Bayesian Analysis2nd ed Springer New York, NY, USA 1985 364 369

[43] Ahmed SE Shrinkage preliminary test estimation in multivariate normal distributions J. Stat. Comput. Sim 1992 43 177 195

